# Supplementary material for: HP1B is a euchromatic Drosophila HP1 homolog with links to metabolism
Source: PLoS One. 2018 Oct 22;13(10):e0205867. doi: 10.1371/journal.pone.0205867 (PMC6197686; doi:10.1371/journal.pone.0205867)
Supplement: S1 Table — (DOCX) [file pone.0205867.s001.docx]

**S1 Table. Genotype and sex strongly impact feeding behavior.**

**A.** Means and standard deviation of food consumption as measured by the CAFÉ assay. Note that the capillaries used in trial 3 had a different diameter than the ones used in trials 1 and 2, and thus, the drop in food levels within the glass capillaries [in mm] is quite different.

**B.** P-values for the comparisons listed in the first column for the three independent trials of the CAFÉ assay that were conducted to estimate food consumption. P-values are derived from a Tukey’s HSD test, unless normality assumption was violated. For non-normal data, a Kruskal-Wallis rank sum test was used (indicated by * in the table below).

**A. Means and standard deviation**

| **Genotype** | **Sex** | **Trial 1** | **Trial 2** | **Trial 3** |
| --- | --- | --- | --- | --- |
| *yw* | combined | 5.71+/-1.98 | 6.31+/-1.76 | 2.33+/-0.72 |
| *HP1b^16^* | combined | 4.12+/-1.62 | 4.00+/-1.09 | 1.77+/-0.72 |
| *HP1b^86^* | combined | 4.65+/-0.81 | 4.63+/-1.84 | 1.68+/-0.70 |
| *yw* | female | 7.15+/-1.72 | 6.37+/-1.10 | 2.56+/-0.65 |
| *HP1b^16^* | female | 5.47+/-0.74 | 4.72+/-0.84 | 2.23+/-0.652 |
| *HP1b^86^* | female | 5.10+/-0.48 | 5.32+/-1.95 | 1.86+/-0.90 |
| *yw* | male | 4.26+/-0.83 | 6.25+/-2.33 | 2.09+/-0.74 |
| *HP1b^16^* | male | 2.76+/-0.83 | 3.28+/-0.81 | 1.31+/-0.45 |
| *HP1b^86^* | male | 4.20+/-0.88 | 3.94+/-1.54 | 1.51+/-0.41 |

**B. P-values**

| **Comparison** | **Sex** | **Trial 1** | **Trial 2** | **Trial 3** |
| --- | --- | --- | --- | --- |
| *yw – HP1b^16^* | combined | **0.0185367** | **0.0003155** | 0.0516329 |
| *yw – HP1b^86^* | combined | 0.1401623 | **0.0095041** | **0.0208996** |
| *yw – HP1b^16^* | female | 0.1489* | 0.0655234 | 0.6461443 |
| *yw – HP1b^86^* | female | 0.08326* | 0.3038136 | 0.1633411 |
| *yw – HP1b^16^* | male | 0.0774069 | **0.002322*** | **0.0271581** |
| *yw – HP1b^86^* | male | 0.9943959 | **0.02742*** | 0.1146129 |
