# Supplementary material for: HP1B is a euchromatic Drosophila HP1 homolog with links to metabolism
Source: PLoS One. 2018 Oct 22;13(10):e0205867. doi: 10.1371/journal.pone.0205867 (PMC6197686; doi:10.1371/journal.pone.0205867)
Supplement: S2 Table — (DOCX) [file pone.0205867.s002.docx]

**S2 Table. Expanded GO analysis results I.** Output of the PANTHER Overrepresentation Test for the PANTHER GO-Slim Biological Process terms (PANTHER version 10.0) for the genes significantly upregulated in both *HP1b* mutant strains. p-values are Bonferroni-corrected. Lower level “child” terms that were omitted in Fig 8 are marked by *. Table entries above the thick line are over-represented in the gene set regulated by HP1B, entries below the line are under-represented.

| PANTHER GO-Slim term -Biological Process | # of genes in genome | Observed # of genes | Expected # of  genes | Fold enrich-ment | p-value |
| --- | --- | --- | --- | --- | --- |
| tricarboxylic acid cycle (GO:0006099) * | 27 | 7 | 1.01 | 6.92 | 1.67E-02 |
| cellular amino acid catabolic process (GO:0009063) * | 49 | 10 | 1.84 | 5.45 | 4.15E-03 |
| oxidative phosphorylation (GO:0006119) * | 55 | 10 | 2.06 | 4.85 | 1.08E-02 |
| phosphate ion transport (GO:0006817) * | 115 | 20 | 4.31 | 4.64 | 5.15E-06 |
| extracellular transport (GO:0006858) | 147 | 25 | 5.51 | 4.54 | 1.48E-07 |
| monosaccharide metabolic process (GO:0005996) * | 122 | 20 | 4.57 | 4.37 | 1.32E-05 |
| carbohydrate transport (GO:0008643) | 111 | 18 | 4.16 | 4.33 | 6.93E-05 |
| generation of precursor metabolites and energy (GO:0006091) | 287 | 45 | 10.75 | 4.18 | 3.66E-13 |
| response to toxic substance (GO:0009636) | 72 | 11 | 2.70 | 4.08 | 2.15E-02 |
| fatty acid metabolic process (GO:0006631) | 122 | 18 | 4.57 | 3.94 | 2.61E-04 |
| respiratory electron transport chain (GO:0022904) | 238 | 33 | 8.92 | 3.70 | 5.19E-08 |
| carbohydrate metabolic process (GO:0005975) | 492 | 65 | 18.44 | 3.53 | 6.33E-16 |
| lipid transport (GO:0006869) | 217 | 28 | 8.13 | 3.44 | 5.33E-06 |
| cellular amino acid metabolic process (GO:0006520) | 235 | 30 | 8.81 | 3.41 | 2.15E-06 |
| anion transport (GO:0006820) * | 144 | 17 | 5.40 | 3.15 | 8.51E-03 |
| lipid metabolic process (GO:0006629) | 693 | 75 | 25.97 | 2.89 | 5.98E-14 |
| steroid metabolic process (GO:0008202) | 159 | 17 | 5.96 | 2.85 | 2.76E-02 |
| ion transport (GO:0006811) | 492 | 52 | 18.44 | 2.82 | 7.42E-09 |
| cation transport (GO:0006812) | 378 | 38 | 14.16 | 2.68 | 1.28E-05 |
| proteolysis (GO:0006508) | 606 | 60 | 22.71 | 2.64 | 2.81E-09 |
| transport (GO:0006810) * | 1409 | 89 | 52.80 | 1.69 | 1.53E-04 |
| localization (GO:0051179) | 1462 | 89 | 54.78 | 1.62 | 6.87E-04 |
| metabolic process (GO:0008152) | 4996 | 295 | 187.21 | 1.58 | 7.08E-20 |
| primary metabolic process (GO:0044238) | 4149 | 233 | 155.47 | 1.50 | 8.54E-11 |
| regulation of biological process (GO:0050789) | 1104 | 19 | 41.37 | .46 | 9.28E-03 |
| nucleobase-containing compound metabolic process (GO:0006139) | 1694 | 25 | 63.48 | .39 | 1.24E-06 |
| regulation of transcription from RNA polymerase II promoter (GO:0006357)* | 487 | 3 | 18.25 | < 0.2 | 2.16E-03 |
| transcription from RNA polymerase II promoter (GO:0006366) * | 686 | 4 | 25.71 | < 0.2 | 1.74E-05 |
| transcription, DNA-dependent (GO:0006351) * | 729 | 4 | 27.32 | < 0.2 | 4.07E-06 |
| regulation of nucleobase-containing compound metabolic process (GO:0019219) * | 594 | 3 | 22.26 | < 0.2 | 5.99E-05 |
| RNA metabolic process (GO:0016070) * | 1039 | 4 | 38.93 | < 0.2 | 7.14E-11 |
| response to stress (GO:0006950) | 285 | 1 | 10.68 | < 0.2 | 4.70E-02 |
