# Supplementary material for: HP1B is a euchromatic Drosophila HP1 homolog with links to metabolism
Source: PLoS One. 2018 Oct 22;13(10):e0205867. doi: 10.1371/journal.pone.0205867 (PMC6197686; doi:10.1371/journal.pone.0205867)
Supplement: S3 Table — (DOCX) [file pone.0205867.s003.docx]

**S3 Table. Expanded GO analysis results II.** Output of the PANTHER Overrepresentation Test for the *GO Biological Process* terms (PANTHER version 10.0; GO Ontology database Released 2016-05-20) for the genes significantly upregulated in both *HP1b* mutant strains. p-values are Bonferroni-corrected. Table entries above the thick line are over-represented in the gene set regulated by HP1B, entries below the line are under-represented.

| GO biological process term – complete set | # of genes in genome | Observed # of genes | Expected # of  genes | Fold enrich-ment | p-value |
| --- | --- | --- | --- | --- | --- |
| cellular acyl-CoA homeostasis (GO:0042049) | 7 | 5 | .26 | 19.06 | 2.23E-02 |
| cellular anion homeostasis (GO:0030002) | 7 | 5 | .26 | 19.06 | 2.23E-02 |
| mitochondrial electron transport, ubiquinol to cytochrome c (GO:0006122) | 14 | 7 | .52 | 13.34 | 3.61E-03 |
| pyruvate metabolic process (GO:0006090) | 36 | 12 | 1.35 | 8.90 | 5.39E-05 |
| single-organism carbohydrate catabolic process (GO:0044724) | 30 | 9 | 1.12 | 8.01 | 7.45E-03 |
| ADP metabolic process (GO:0046031) | 27 | 8 | 1.01 | 7.91 | 2.91E-02 |
| ribonucleoside diphosphate metabolic process (GO:0009185) | 27 | 8 | 1.01 | 7.91 | 2.91E-02 |
| purine ribonucleoside diphosphate metabolic process (GO:0009179) | 27 | 8 | 1.01 | 7.91 | 2.91E-02 |
| purine nucleoside diphosphate metabolic process (GO:0009135) | 27 | 8 | 1.01 | 7.91 | 2.91E-02 |
| mitochondrial ATP synthesis coupled electron transport (GO:0042775) | 71 | 21 | 2.66 | 7.89 | 2.59E-09 |
| ATP synthesis coupled electron transport (GO:0042773) | 75 | 22 | 2.81 | 7.83 | 8.74E-10 |
| electron transport chain (GO:0022900) | 89 | 26 | 3.34 | 7.80 | 6.68E-12 |
| respiratory electron transport chain (GO:0022904) | 84 | 24 | 3.15 | 7.62 | 1.27E-10 |
| oxidative phosphorylation (GO:0006119) | 81 | 23 | 3.04 | 7.58 | 4.88E-10 |
| carbohydrate catabolic process (GO:0016052) | 32 | 9 | 1.20 | 7.51 | 1.25E-02 |
| cellular lipid catabolic process (GO:0044242) | 47 | 13 | 1.76 | 7.38 | 1.20E-04 |
| energy derivation by oxidation of organic compounds (GO:0015980) | 133 | 36 | 4.98 | 7.22 | 2.93E-16 |
| generation of precursor metabolites and energy (GO:0006091) | 169 | 45 | 6.33 | 7.11 | 1.26E-20 |
| mitochondrial electron transport, NADH to ubiquinone (GO:0006120) | 34 | 9 | 1.27 | 7.06 | 2.02E-02 |
| ATP metabolic process (GO:0046034) | 133 | 34 | 4.98 | 6.82 | 1.69E-14 |
| cellular respiration (GO:0045333) | 122 | 31 | 4.57 | 6.78 | 5.88E-13 |
| purine ribonucleoside monophosphate metabolic process (GO:0009167) | 145 | 35 | 5.43 | 6.44 | 3.08E-14 |
| purine nucleoside monophosphate metabolic process (GO:0009126) | 145 | 35 | 5.43 | 6.44 | 3.08E-14 |
| purine ribonucleoside triphosphate metabolic process (GO:0009205) | 143 | 34 | 5.36 | 6.34 | 1.41E-13 |
| purine nucleoside triphosphate metabolic process (GO:0009144) | 143 | 34 | 5.36 | 6.34 | 1.41E-13 |
| ribonucleoside triphosphate metabolic process (GO:0009199) | 144 | 34 | 5.40 | 6.30 | 1.73E-13 |
| nicotinamide nucleotide metabolic process (GO:0046496) | 43 | 10 | 1.61 | 6.21 | 1.94E-02 |
| pyridine nucleotide metabolic process (GO:0019362) | 43 | 10 | 1.61 | 6.21 | 1.94E-02 |
| ribonucleoside monophosphate metabolic process (GO:0009161) | 151 | 35 | 5.66 | 6.19 | 1.04E-13 |
| nucleoside triphosphate metabolic process (GO:0009141) | 147 | 34 | 5.51 | 6.17 | 3.15E-13 |
| nucleoside monophosphate metabolic process (GO:0009123) | 152 | 35 | 5.70 | 6.14 | 1.26E-13 |
| lipid localization (GO:0010876) | 51 | 11 | 1.91 | 5.76 | 1.38E-02 |
| purine ribonucleoside metabolic process (GO:0046128) | 171 | 36 | 6.41 | 5.62 | 6.81E-13 |
| monocarboxylic acid metabolic process (GO:0032787) | 143 | 30 | 5.36 | 5.60 | 2.41E-10 |
| pyridine-containing compound metabolic process (GO:0072524) | 48 | 10 | 1.80 | 5.56 | 4.94E-02 |
| purine nucleoside metabolic process (GO:0042278) | 174 | 36 | 6.52 | 5.52 | 1.15E-12 |
| ribonucleoside metabolic process (GO:0009119) | 179 | 36 | 6.71 | 5.37 | 2.69E-12 |
| nucleoside metabolic process (GO:0009116) | 192 | 37 | 7.19 | 5.14 | 3.92E-12 |
| hydrogen transport (GO:0006818) | 63 | 12 | 2.36 | 5.08 | 1.80E-02 |
| glycosyl compound metabolic process (GO:1901657) | 196 | 37 | 7.34 | 5.04 | 7.33E-12 |
| purine nucleotide metabolic process (GO:0006163) | 192 | 36 | 7.19 | 5.00 | 2.15E-11 |
| purine ribonucleotide metabolic process (GO:0009150) | 189 | 35 | 7.08 | 4.94 | 7.32E-11 |
| lipid catabolic process (GO:0016042) | 76 | 14 | 2.85 | 4.92 | 4.59E-03 |
| ribonucleotide metabolic process (GO:0009259) | 194 | 35 | 7.27 | 4.81 | 1.54E-10 |
| ribose phosphate metabolic process (GO:0019693) | 200 | 36 | 7.49 | 4.80 | 7.11E-11 |
| small molecule catabolic process (GO:0044282) | 85 | 15 | 3.19 | 4.71 | 3.32E-03 |
| purine-containing compound metabolic process (GO:0072521) | 214 | 37 | 8.02 | 4.61 | 1.02E-10 |
| nucleotide metabolic process (GO:0009117) | 243 | 41 | 9.11 | 4.50 | 9.04E-12 |
| fatty acid metabolic process (GO:0006631) | 83 | 14 | 3.11 | 4.50 | 1.24E-02 |
| nucleoside phosphate metabolic process (GO:0006753) | 246 | 41 | 9.22 | 4.45 | 1.35E-11 |
| organic anion transport (GO:0015711) | 91 | 15 | 3.41 | 4.40 | 7.54E-03 |
| coenzyme metabolic process (GO:0006732) | 99 | 16 | 3.71 | 4.31 | 4.54E-03 |
| single-organism catabolic process (GO:0044712) | 211 | 33 | 7.91 | 4.17 | 3.49E-08 |
| inorganic ion transmembrane transport (GO:0098660) | 96 | 15 | 3.60 | 4.17 | 1.42E-02 |
| oxidation-reduction process (GO:0055114) | 551 | 86 | 20.65 | 4.17 | 2.13E-25 |
| chitin metabolic process (GO:0006030) | 117 | 18 | 4.38 | 4.11 | 2.07E-03 |
| inorganic cation transmembrane transport (GO:0098662) | 92 | 14 | 3.45 | 4.06 | 3.88E-02 |
| cation transmembrane transport (GO:0098655) | 92 | 14 | 3.45 | 4.06 | 3.88E-02 |
| nucleobase-containing small molecule metabolic process (GO:0055086) | 284 | 43 | 10.64 | 4.04 | 7.27E-11 |
| carboxylic acid metabolic process (GO:0019752) | 334 | 50 | 12.52 | 3.99 | 7.89E-13 |
| anion transport (GO:0006820) | 124 | 18 | 4.65 | 3.87 | 4.64E-03 |
| glucosamine-containing compound metabolic process (GO:1901071) | 125 | 18 | 4.68 | 3.84 | 5.18E-03 |
| amino sugar metabolic process (GO:0006040) | 126 | 18 | 4.72 | 3.81 | 5.78E-03 |
| organic acid metabolic process (GO:0006082) | 351 | 50 | 13.15 | 3.80 | 5.32E-12 |
| oxoacid metabolic process (GO:0043436) | 351 | 50 | 13.15 | 3.80 | 5.32E-12 |
| ion transmembrane transport (GO:0034220) | 133 | 18 | 4.98 | 3.61 | 1.20E-02 |
| aminoglycan metabolic process (GO:0006022) | 141 | 19 | 5.28 | 3.60 | 6.99E-03 |
| small molecule metabolic process (GO:0044281) | 738 | 94 | 27.65 | 3.40 | 1.04E-21 |
| organophosphate metabolic process (GO:0019637) | 366 | 46 | 13.71 | 3.35 | 5.54E-09 |
| carbohydrate derivative metabolic process (GO:1901135) | 526 | 66 | 19.71 | 3.35 | 6.01E-14 |
| transmembrane transport (GO:0055085) | 457 | 49 | 17.12 | 2.86 | 2.48E-07 |
| carbohydrate metabolic process (GO:0005975) | 282 | 29 | 10.57 | 2.74 | 4.31E-03 |
| lipid metabolic process (GO:0006629) | 375 | 34 | 14.05 | 2.42 | 8.54E-03 |
| ion transport (GO:0006811) | 395 | 35 | 14.80 | 2.36 | 1.00E-02 |
| organonitrogen compound metabolic process (GO:1901564) | 944 | 82 | 35.37 | 2.32 | 4.09E-09 |
| single-organism metabolic process (GO:0044710) | 1846 | 145 | 69.17 | 2.10 | 3.78E-15 |
| proteolysis (GO:0006508) | 716 | 53 | 26.83 | 1.98 | 7.09E-03 |
| phosphorus metabolic process (GO:0006793) | 779 | 57 | 29.19 | 1.95 | 4.11E-03 |
| phosphate-containing compound metabolic process (GO:0006796) | 765 | 53 | 28.67 | 1.85 | 4.42E-02 |
| metabolic process (GO:0008152) | 4314 | 260 | 161.66 | 1.61 | 4.55E-16 |
| organic substance metabolic process (GO:0071704) | 3834 | 205 | 143.67 | 1.43 | 1.01E-05 |
| single-organism process (GO:0044699) | 6316 | 285 | 236.68 | 1.20 | 3.21E-02 |
| Unclassified (UNCLASSIFIED) | 2707 | 86 | 101.44 | .85 | 0.00E00 |
| system development (GO:0048731) | 2274 | 48 | 85.21 | .56 | 4.38E-03 |
| biological regulation (GO:0065007) | 3612 | 75 | 135.35 | .55 | 2.74E-07 |
| cell differentiation (GO:0030154) | 2240 | 46 | 83.94 | .55 | 2.23E-03 |
| cellular developmental process (GO:0048869) | 2316 | 47 | 86.79 | .54 | 9.21E-04 |
| anatomical structure morphogenesis (GO:0009653) | 1719 | 31 | 64.42 | .48 | 2.28E-03 |
| animal organ development (GO:0048513) | 1325 | 23 | 49.65 | .46 | 2.44E-02 |
| regulation of biological process (GO:0050789) | 3280 | 51 | 122.91 | .41 | 8.41E-13 |
| regulation of metabolic process (GO:0019222) | 1693 | 26 | 63.44 | .41 | 4.91E-05 |
| response to stimulus (GO:0050896) | 2420 | 37 | 90.68 | .41 | 1.34E-08 |
| nervous system development (GO:0007399) | 1590 | 24 | 59.58 | .40 | 9.66E-05 |
| cellular component organization (GO:0016043) | 2468 | 37 | 92.48 | .40 | 3.58E-09 |
| neurogenesis (GO:0022008) | 1424 | 21 | 53.36 | .39 | 3.48E-04 |
| cellular component organization or biogenesis (GO:0071840) | 2526 | 37 | 94.66 | .39 | 7.07E-10 |
| cell development (GO:0048468) | 1446 | 21 | 54.19 | .39 | 1.97E-04 |
| regulation of cellular metabolic process (GO:0031323) | 1586 | 23 | 59.43 | .39 | 3.90E-05 |
| regulation of primary metabolic process (GO:0080090) | 1543 | 22 | 57.82 | .38 | 4.31E-05 |
| regulation of cellular process (GO:0050794) | 3061 | 43 | 114.70 | .37 | 8.60E-14 |
| single-organism organelle organization (GO:1902589) | 1141 | 16 | 42.76 | .37 | 3.21E-03 |
| organelle organization (GO:0006996) | 1627 | 22 | 60.97 | .36 | 4.45E-06 |
| multi-organism process (GO:0051704) | 1304 | 17 | 48.86 | .35 | 1.21E-04 |
| multicellular organism reproduction (GO:0032504) | 1198 | 15 | 44.89 | .33 | 2.31E-04 |
| reproduction (GO:0000003) | 1375 | 17 | 51.52 | .33 | 1.68E-05 |
| cellular process involved in reproduction in multicellular organism (GO:0022412) | 809 | 10 | 30.32 | .33 | 3.19E-02 |
| single organism reproductive process (GO:0044702) | 1037 | 12 | 38.86 | .31 | 5.99E-04 |
| gamete generation (GO:0007276) | 870 | 10 | 32.60 | .31 | 5.88E-03 |
| cellular component assembly (GO:0022607) | 796 | 9 | 29.83 | .30 | 1.40E-02 |
| positive regulation of biological process (GO:0048518) | 1160 | 13 | 43.47 | .30 | 6.06E-05 |
| cellular macromolecule biosynthetic process (GO:0034645) | 723 | 8 | 27.09 | .30 | 3.21E-02 |
| reproductive process (GO:0022414) | 1177 | 13 | 44.11 | .29 | 3.69E-05 |
| multicellular organismal reproductive process (GO:0048609) | 999 | 11 | 37.44 | .29 | 5.12E-04 |
| macromolecule biosynthetic process (GO:0009059) | 731 | 8 | 27.39 | .29 | 2.55E-02 |
| regulation of response to stimulus (GO:0048583) | 914 | 10 | 34.25 | .29 | 1.68E-03 |
| multi-organism reproductive process (GO:0044703) | 1006 | 11 | 37.70 | .29 | 4.18E-04 |
| sexual reproduction (GO:0019953) | 1006 | 11 | 37.70 | .29 | 4.18E-04 |
| organ morphogenesis (GO:0009887) | 841 | 9 | 31.51 | .29 | 3.85E-03 |
| negative regulation of biological process (GO:0048519) | 1147 | 12 | 42.98 | .28 | 2.43E-05 |
| cellular component biogenesis (GO:0044085) | 868 | 9 | 32.53 | .28 | 1.75E-03 |
| regulation of developmental process (GO:0050793) | 679 | 7 | 25.44 | .28 | 3.17E-02 |
| negative regulation of cellular process (GO:0048523) | 1019 | 10 | 38.18 | .26 | 7.54E-05 |
| macromolecular complex subunit organization (GO:0043933) | 936 | 9 | 35.07 | .26 | 2.31E-04 |
| oogenesis (GO:0048477) | 639 | 6 | 23.94 | .25 | 2.68E-02 |
| cell communication (GO:0007154) | 1408 | 13 | 52.76 | .25 | 3.20E-08 |
| regulation of signal transduction (GO:0009966) | 651 | 6 | 24.39 | .25 | 1.87E-02 |
| regulation of multicellular organismal process (GO:0051239) | 661 | 6 | 24.77 | .24 | 1.38E-02 |
| cell projection organization (GO:0030030) | 682 | 6 | 25.56 | .23 | 7.30E-03 |
| cell cycle process (GO:0022402) | 682 | 6 | 25.56 | .23 | 7.30E-03 |
| neuron development (GO:0048666) | 686 | 6 | 25.71 | .23 | 6.46E-03 |
| regulation of macromolecule metabolic process (GO:0060255) | 1498 | 13 | 56.13 | .23 | 1.78E-09 |
| positive regulation of cellular process (GO:0048522) | 1039 | 9 | 38.93 | .23 | 9.64E-06 |
| positive regulation of cellular metabolic process (GO:0031325) | 578 | 5 | 21.66 | .23 | 4.09E-02 |
| cell morphogenesis (GO:0000902) | 702 | 6 | 26.31 | .23 | 3.95E-03 |
| protein modification process (GO:0036211) | 956 | 8 | 35.82 | .22 | 2.90E-05 |
| cellular protein modification process (GO:0006464) | 956 | 8 | 35.82 | .22 | 2.90E-05 |
| cell projection morphogenesis (GO:0048858) | 605 | 5 | 22.67 | .22 | 1.78E-02 |
| movement of cell or subcellular component (GO:0006928) | 612 | 5 | 22.93 | .22 | 1.43E-02 |
| cell part morphogenesis (GO:0032990) | 613 | 5 | 22.97 | .22 | 1.39E-02 |
| positive regulation of metabolic process (GO:0009893) | 613 | 5 | 22.97 | .22 | 1.39E-02 |
| cell cycle (GO:0007049) | 736 | 6 | 27.58 | .22 | 1.37E-03 |
| regulation of signaling (GO:0023051) | 737 | 6 | 27.62 | .22 | 1.33E-03 |
| regulation of cell communication (GO:0010646) | 752 | 6 | 28.18 | .21 | 8.30E-04 |
| macromolecule modification (GO:0043412) | 1011 | 8 | 37.88 | .21 | 5.02E-06 |
| generation of neurons (GO:0048699) | 910 | 7 | 34.10 | .21 | 2.62E-05 |
| single organism signaling (GO:0044700) | 1312 | 10 | 49.16 | .20 | 6.82E-09 |
| signaling (GO:0023052) | 1314 | 10 | 49.24 | .20 | 6.38E-09 |
| neuron differentiation (GO:0030182) | 789 | 6 | 29.57 | .20 | 2.56E-04 |
| cellular macromolecule metabolic process (GO:0044260) | 2245 | 17 | 84.13 | .20 | 9.79E-18 |
| regulation of biosynthetic process (GO:0009889) | 1074 | 8 | 40.25 | < 0.2 | 6.48E-07 |
| positive regulation of macromolecule metabolic process (GO:0010604) | 547 | 4 | 20.50 | < 0.2 | 2.33E-02 |
| regulation of cellular biosynthetic process (GO:0031326) | 1073 | 7 | 40.21 | < 0.2 | 1.21E-07 |
| regionalization (GO:0003002) | 473 | 3 | 17.72 | < 0.2 | 4.84E-02 |
| cellular protein metabolic process (GO:0044267) | 1424 | 9 | 53.36 | < 0.2 | 2.63E-11 |
| cellular response to stimulus (GO:0051716) | 1444 | 9 | 54.11 | < 0.2 | 1.31E-11 |
| regulation of gene expression (GO:0010468) | 1149 | 7 | 43.06 | < 0.2 | 9.08E-09 |
| regulation of nitrogen compound metabolic process (GO:0051171) | 1152 | 7 | 43.17 | < 0.2 | 8.19E-09 |
| regulation of cellular macromolecule biosynthetic process (GO:2000112) | 1019 | 6 | 38.18 | < 0.2 | 1.24E-07 |
| regulation of macromolecule biosynthetic process (GO:0010556) | 1022 | 6 | 38.30 | < 0.2 | 1.11E-07 |
| negative regulation of macromolecule metabolic process (GO:0010605) | 541 | 3 | 20.27 | < 0.2 | 5.10E-03 |
| organelle fission (GO:0048285) | 425 | 2 | 15.93 | < 0.2 | 3.90E-02 |
| regulation of RNA biosynthetic process (GO:2001141) | 898 | 4 | 33.65 | < 0.2 | 1.62E-07 |
| regulation of transcription, DNA-templated (GO:0006355) | 898 | 4 | 33.65 | < 0.2 | 1.62E-07 |
| regulation of nucleic acid-templated transcription (GO:1903506) | 898 | 4 | 33.65 | < 0.2 | 1.62E-07 |
| regulation of RNA metabolic process (GO:0051252) | 976 | 4 | 36.57 | < 0.2 | 9.96E-09 |
| regulation of nucleobase-containing compound metabolic process (GO:0019219) | 1026 | 4 | 38.45 | < 0.2 | 1.63E-09 |
| chromosome organization (GO:0051276) | 610 | 2 | 22.86 | < 0.2 | 5.92E-05 |
| gene expression (GO:0010467) | 925 | 3 | 34.66 | < 0.2 | 6.52E-09 |
| positive regulation of gene expression (GO:0010628) | 369 | 1 | 13.83 | < 0.2 | 3.39E-02 |
| negative regulation of nitrogen compound metabolic process (GO:0051172) | 373 | 1 | 13.98 | < 0.2 | 2.93E-02 |
| chromatin organization (GO:0006325) | 380 | 1 | 14.24 | < 0.2 | 2.28E-02 |
| negative regulation of gene expression (GO:0010629) | 414 | 1 | 15.51 | < 0.2 | 6.67E-03 |
| nucleic acid metabolic process (GO:0090304) | 913 | 2 | 34.21 | < 0.2 | 8.10E-10 |
| regulation of transcription from RNA polymerase II promoter (GO:0006357) | 495 | 1 | 18.55 | < 0.2 | 3.44E-04 |
| embryo development (GO:0009790) | 505 | 1 | 18.92 | < 0.2 | 2.38E-04 |
| RNA metabolic process (GO:0016070) | 680 | 1 | 25.48 | < 0.2 | 3.38E-07 |
| signal transduction (GO:0007165) | 959 | 1 | 35.94 | < 0.2 | 7.12E-12 |
