# Supplementary material for: HP1B is a euchromatic Drosophila HP1 homolog with links to metabolism
Source: PLoS One. 2018 Oct 22;13(10):e0205867. doi: 10.1371/journal.pone.0205867 (PMC6197686; doi:10.1371/journal.pone.0205867)
Supplement: S4 Table — (DOCX) [file pone.0205867.s004.docx]

**S4 Table. Expanded GO analysis results III.** Output of the PANTHER Overrepresentation Test for the *GO Biological Process* terms (PANTHER version 10.0; GO Ontology database Released 2016-05-20) for the genes significantly downregulated in both *HP1b* mutant strains. p-values are Bonferroni-corrected. Table entries above the thick line are over-represented in the gene set regulated by HP1B, entries below the line are under-represented.

| GO biological process term – complete set | # of genes in genome | Observed # of genes | Expected # of  genes | Fold enrich-ment | p-value |
| --- | --- | --- | --- | --- | --- |
| phenol-containing compound biosynthetic process (GO:0046189) | 21 | 4 | .13 | 31.80 | 2.40E-02 |
| cuticle pigmentation (GO:0048067) | 23 | 4 | .14 | 29.03 | 3.42E-02 |
| organic hydroxy compound biosynthetic process (GO:1901617) | 48 | 6 | .29 | 20.87 | 1.41E-03 |
| developmental pigmentation (GO:0048066) | 75 | 6 | .45 | 13.36 | 1.81E-02 |
| pigmentation (GO:0043473) | 83 | 6 | .50 | 12.07 | 3.19E-02 |
| response to temperature stimulus (GO:0009266) | 130 | 7 | .78 | 8.99 | 3.87E-02 |
| organic hydroxy compound metabolic process (GO:1901615) | 130 | 7 | .78 | 8.99 | 3.87E-02 |
| Unclassified (UNCLASSIFIED) | 2707 | 9 | 16.21 | .56 | 0.00E00 |
