# Supplementary figures and images for: HP1B is a euchromatic Drosophila HP1 homolog with links to metabolism
Source: PLoS One. 2018 Oct 22;13(10):e0205867. doi: 10.1371/journal.pone.0205867 (PMC6197686; doi:10.1371/journal.pone.0205867)

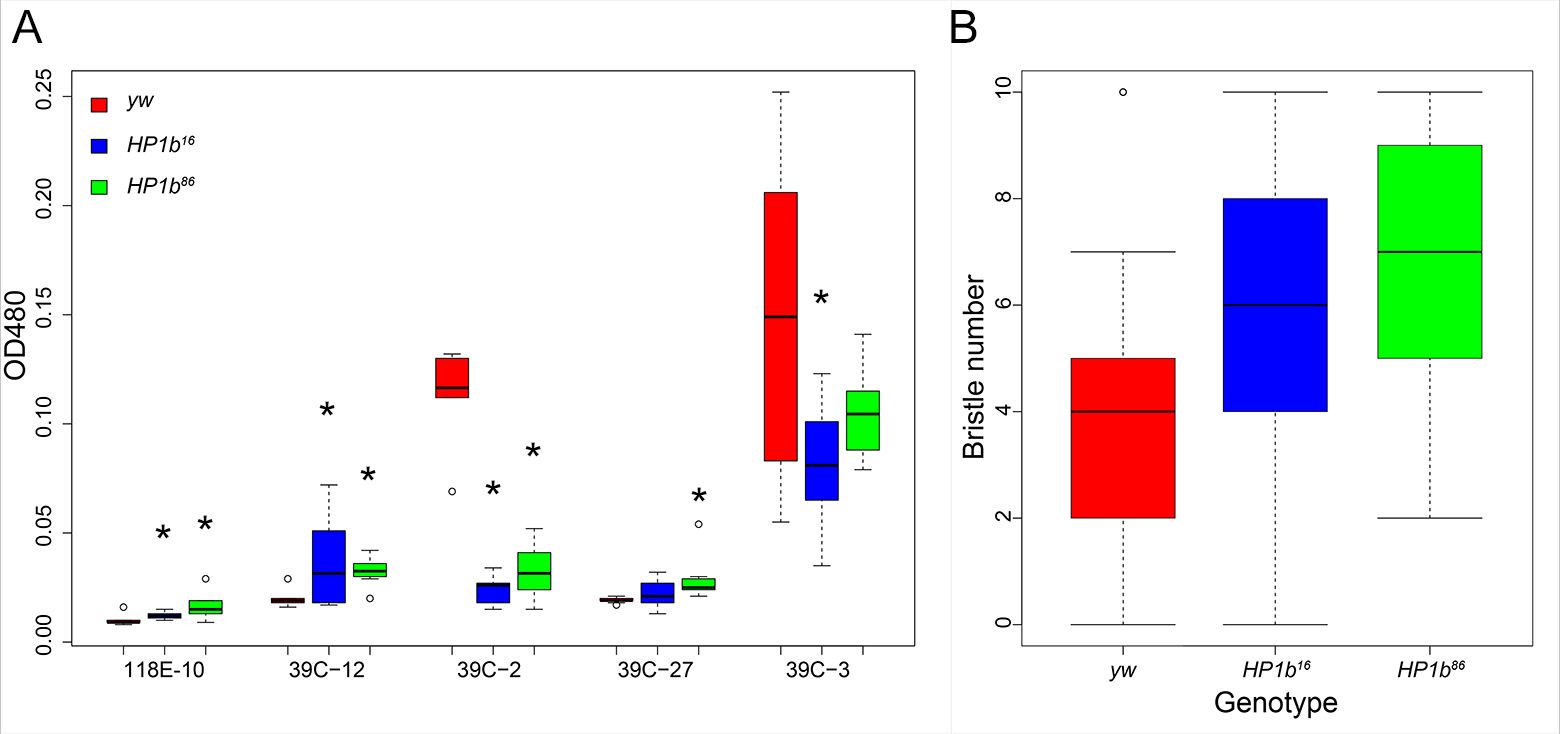

Supplement: S1 Fig — A. Eye pigment assays for females from the five reporter insertions assayed. The response of an hsp70-white reporter to a single mutant HP1b allele in female flies depends on the reporter location. *: comparisons of mutant eye pigment levels to the y-w- control that are statistically significant (p<0.05, t-test). Y-axis: OD480 measuring eye pigment. X-axis: reporter insertions. Results from the y-w- control are in red, from HP1b16 in blue, and from HP1b86 in green. Box plots: black bar—median; box—+25% and -25% quartiles; whiskers—maximum and minimum; circles—outliers; n = 6–10. B. Stubble variegation assays indicate that HP1b is an enhancer of variegation, as the presence of the HP1b mutant alleles significantly increases the number of wildtype bristles compared to the y-w- control (p<0.01, Tukey multiple comparisons of means; n = 19–39). Data from females. Results from the y-w- control are in red, from HP1b16 in blue, and from HP1b86 in green. Box plots: black bar—median; box—+25% and -25% quartiles; whiskers—maximum and minimum; circles—outliers. (TIF) [file pone.0205867.s005.tif]

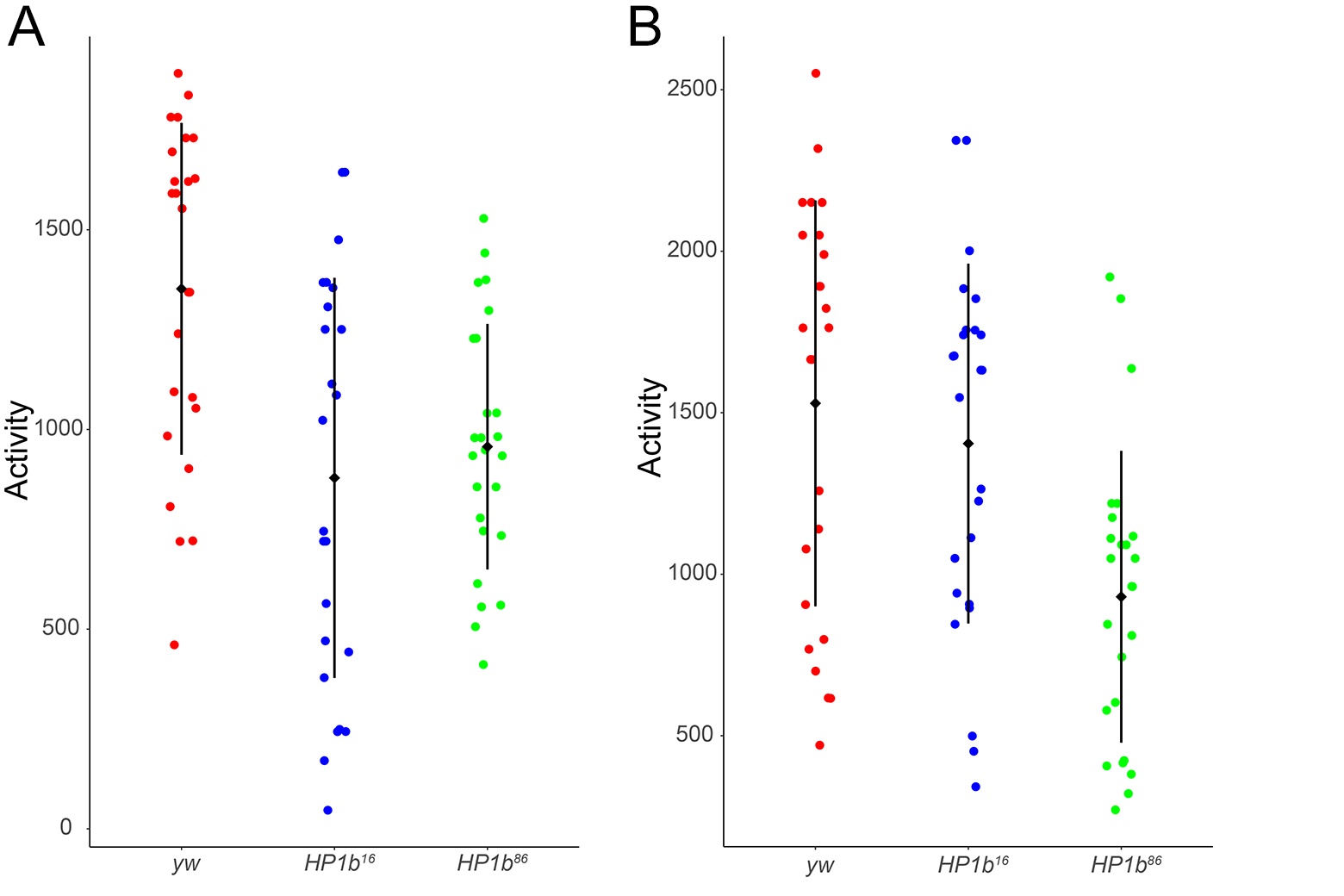

Supplement: S2 Fig — A. Female flies lacking HP1B show significantly lower activity levels than animals of the yw control genotype (p<0.001 for both comparisons; Tukey’s HSD). B. In males, the activity levels of animals lacking HP1B is reduced compared to yw, but the effect is less pronounced than in females and only significant for the HP1b86 allele (p-value not significant for HP1b16, and p<0.001 for HP1b86, Tukey’s HSD). Y-axis: activity level as measured by recorded beam crossings in an activity monitor for 10 flies within the 2hr assay period. Black diamond and bar: mean +/- SD. N = 25 per sex/genotype combination. (TIF) [file pone.0205867.s006.tif]

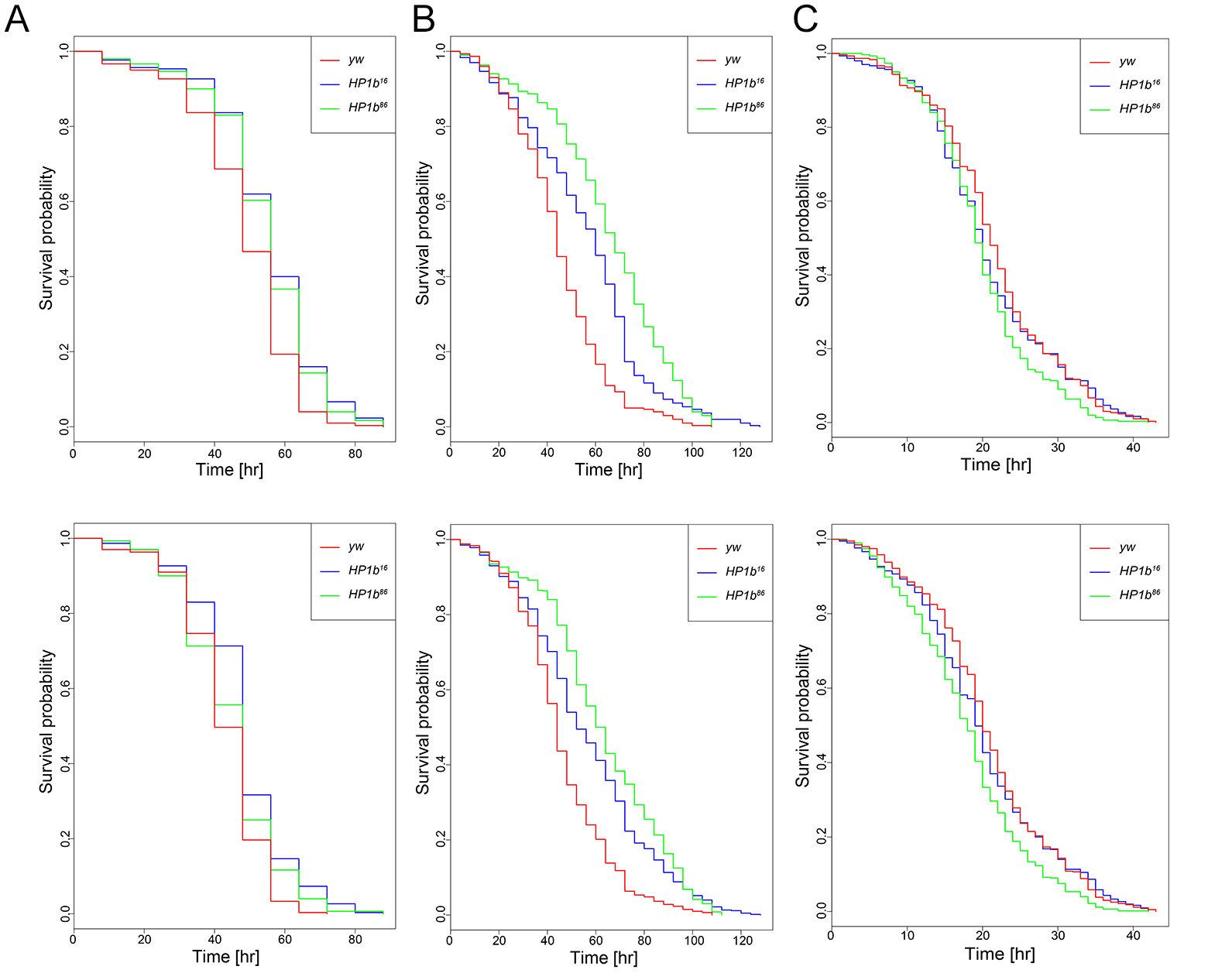

Supplement: S3 Fig — A. Female HP1b mutant animals survive significantly longer during starvation condition than yw control animals (top; p = 6.455e-09 and p = 4.672e-07 for HP1b16 and HP1b86 respectively; Kruskal-Wallis rank sum test), while for males, only the comparison between HP1b16 and yw is significant (bottom; p = 2.054e-07; Kruskal-Wallis rank sum test). B. Female HP1b mutant animals survive significantly longer after exposure to the oxidizer paraquat than yw control animals (top; p = 3.752e-10 and p < 2.2e-16 for HP1b16 and HP1b86 respectively; Kruskal-Wallis rank sum test), as do males (bottom; p = 1.252e-05 and p = 1.79e-14 for HP1b16 and HP1b86 respectively; Kruskal-Wallis rank sum test). C. HP1b mutant animals, both male and female, do not show improved survival during heat stress conditions (37°C) compared to yw control animals. In contrast, HP1b86 shows significantly lower survival (p = 0.001803 in females [top] and p = 1.499e-08in males [bottom]; Kruskal-Wallis rank sum test). For A-C: X-axis—time to death in hours. Y-axis—survival probability. Data shown are from three trials, each with 100 animals per genotype. (TIF) [file pone.0205867.s007.tif]

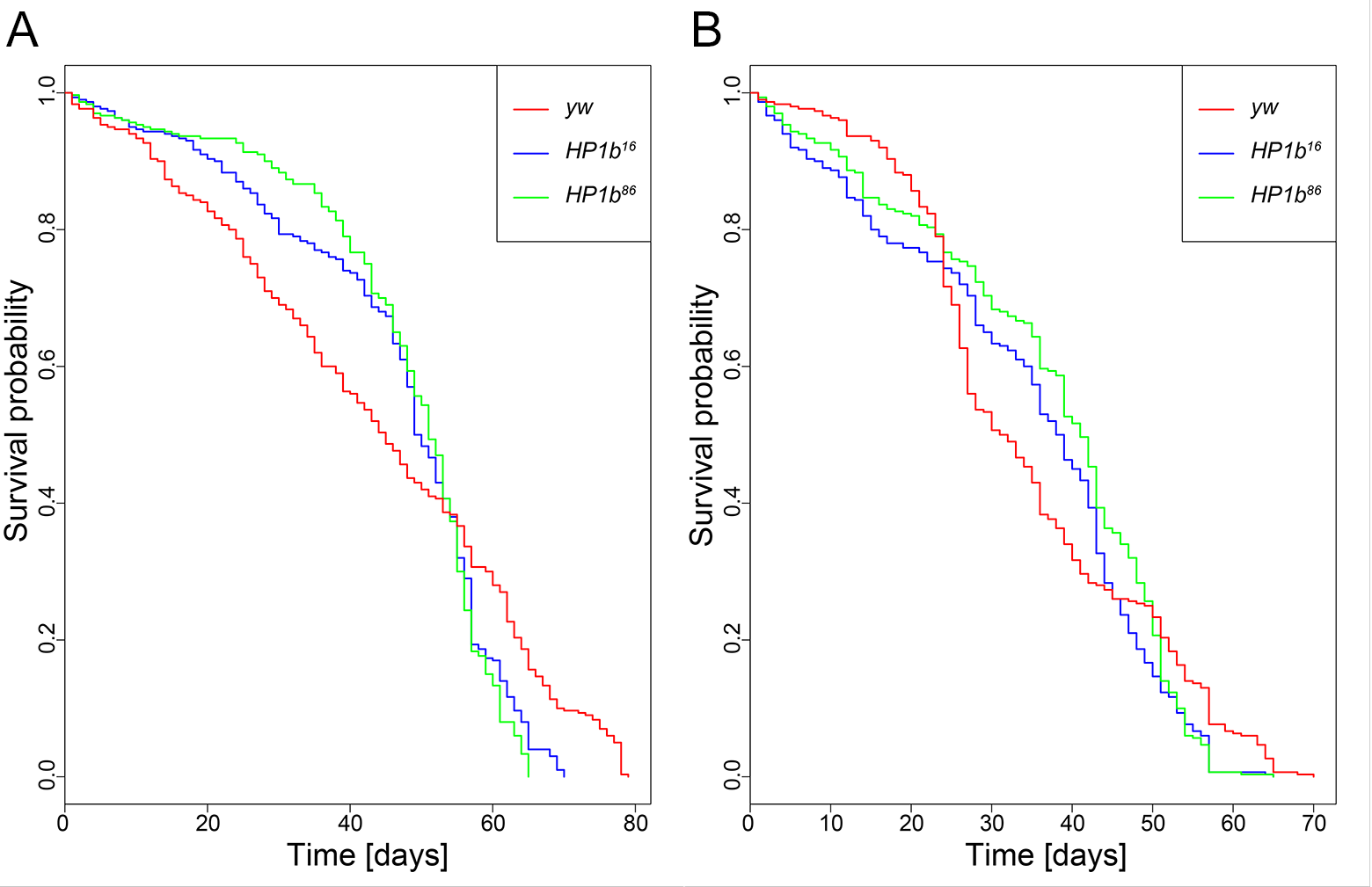

Supplement: S4 Fig — A. Survival curves differ significantly between the HP1b mutant strains and the yw control strain in females (HP1b16: p = 0.0259; HP1b86: p = 0.00563; log rank test). B. In males, the survival curves of HP1b mutant animals do not differ significantly from the survival curves of yw control animals (p not significant; log rank test). A+B: X-axis—time to death in days. Y-axis—survival probability. Data shown are combined from three trials, each with 100 animals per genotype/sex. (TIF) [file pone.0205867.s008.tif]

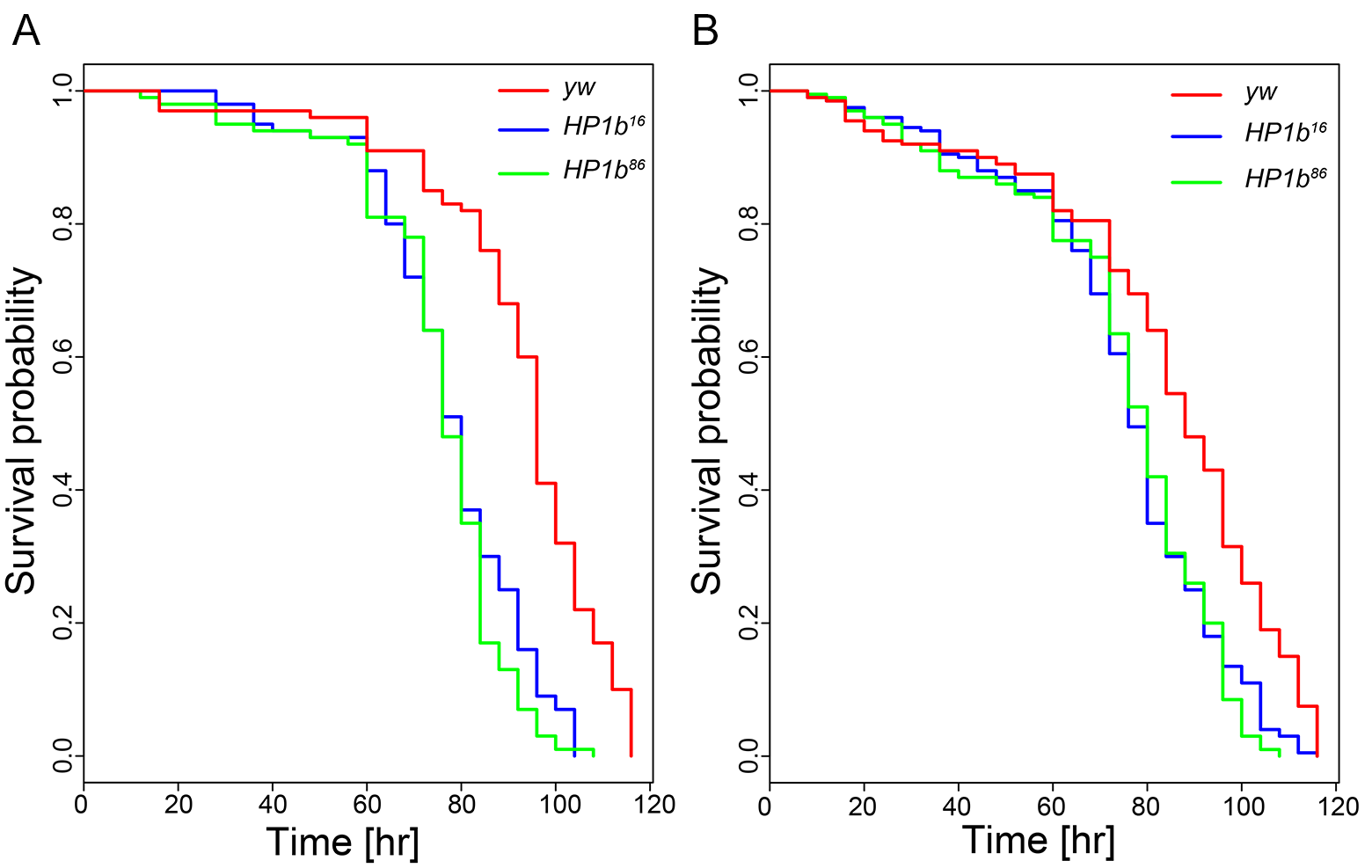

Supplement: S5 Fig — The paraquat concentration was adjusted to take into account differences in feeding behavior between HP1b mutants (blue, green) and the yw control strain (red). A. Females. After adjusting the paraquat concentration, female HP1b mutant animals die earlier than their yw counterparts (HP1b16: p = 7.413e-11; HP1b86: p = 2.113e-14; Kruskal-Wallis rank sum test). B. Males. After adjusting the paraquat concentration, no difference in survival is detected between HP1b mutants and yw (HP1b16: p = 0.31; HP1b86: p = 0.9883; Kruskal-Wallis-rank sum test). Y-axis: survival probability; X-axis: Time to death in hours. N = 100 animals per genotype/sex. (TIF) [file pone.0205867.s009.tif]

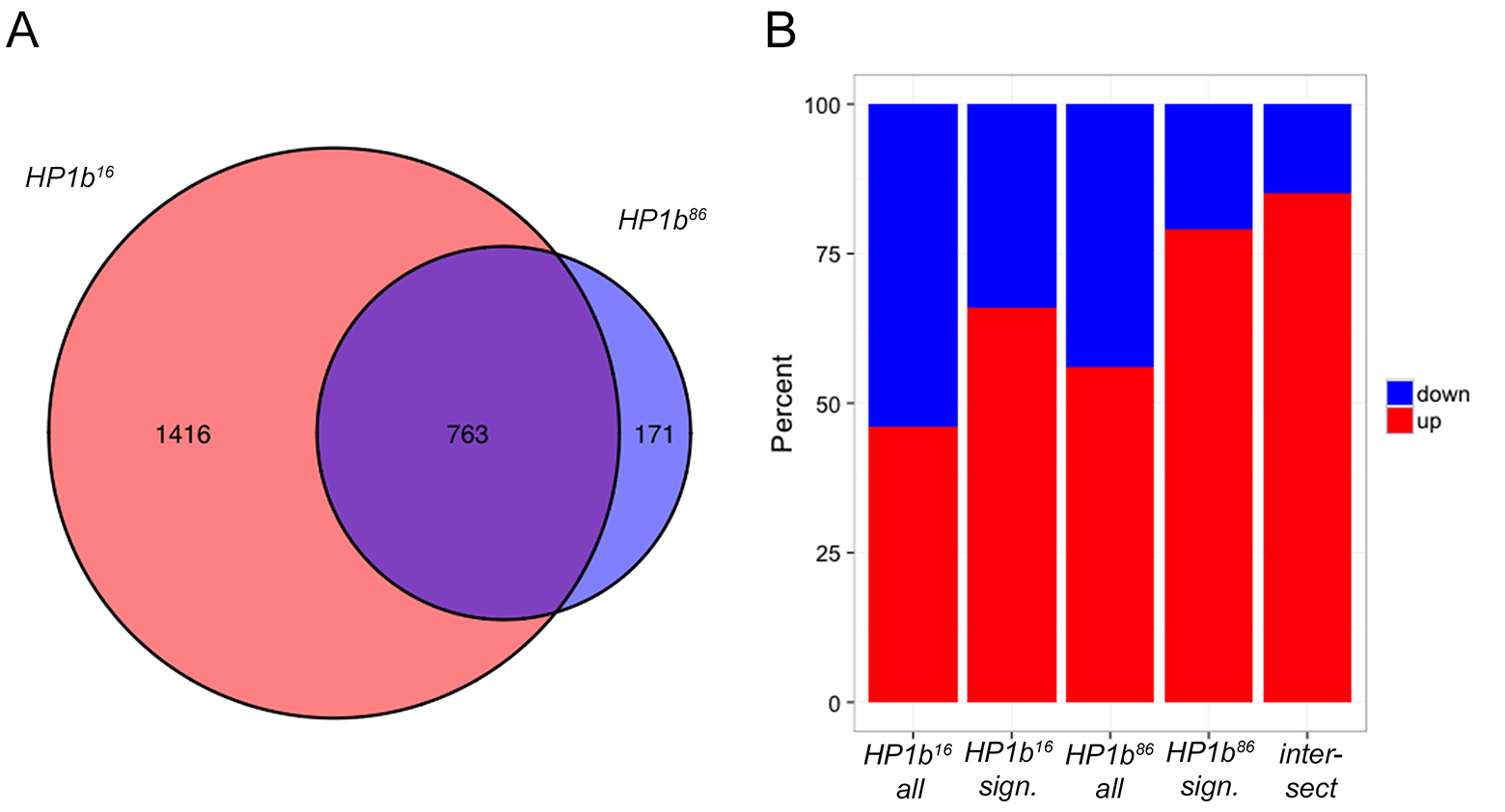

Supplement: S6 Fig — A. Venn diagram illustrating the overlap in the gene sets identified as significantly altered in gene expression in the HP1b16 and HP1b86 mutant strains. Only genes with an FDR<0.05 are included (Benjamini-Hochberg). B. Stacked bar graph illustrating the percentage of genes up- versus down-regulated upon loss of HP1B compared to the yw control strain. Red: upregulated genes; blue: downregulated genes. (TIF) [file pone.0205867.s010.tif]
